# Regulation of Fear Memory by Glucocorticoid and Cholinergic Receptors within the Dorsal Striatum

**DOI:** 10.3389/fnbeh.2012.00042

**Published:** 2012-06-19

**Authors:** Rafael Roesler

**Affiliations:** ^1^Laboratory of Neuropharmacology and Neural Tumor Biology, Department of Pharmacology, Institute for Basic Health Sciences, Federal University of Rio Grande do SulPorto Alegre, Brazil; ^2^Cancer Research Laboratory, University Hospital Research Center, Federal University of Rio Grande do SulPorto Alegre, Brazil; ^3^National Institute for Translational MedicinePorto Alegre, Brazil

A prevailing view regarding the systems neurobiology of memory formation is that different types of memory are mediated by relatively independent brain systems. In particular, the early phase of processing of declarative memories is thought to rely mostly on the dorsal hippocampus and related temporal lobe areas, while the dorsal striatum is proposed to mediate the formation of memories for procedures and habits. For example, pharmacological stimulation of the dorsal hippocampus enhances memory in water maze and radial maze tasks when training protocols based on spatial memory are used, whereas stimulation of the dorsal striatum enhances procedural versions of these tasks that use visible cues during training (Packard and White, 1991; Packard and Teather, 1998; for a recent review, see Packard and Goodman, 2012).

The consolidation of memory for inhibitory avoidance (IA), a widely used model of fear-motivated conditioning, crucially depends on neurotransmitter and protein kinase signaling, protein synthesis, and gene expression in the dorsal hippocampus (Izquierdo and Medina, 1997). In IA training, rats or mice learn, typically after a single-trial, to associate a particular location in a training box (a grid floor or a dark compartment) with an aversive stimulus (a mild footshock). Although several authors have considered IA an instrumental learning task, in which the animal learns to avoid the behavior of stepping down or stepping through to the shock compartment (Wilensky et al., 2000), the available evidence indicates that IA can be best described as a type of contextual fear conditioning (CFC), in which a novel context (an area within the training box) is associated with a conditioned stimulus (CS), i.e., a footshock. Consistent with this view, formation of memory for avoiding the footshock compartment requires contextual information, but not instrumental or procedural learning during training (Vazdarjanova and McGaugh, 1998; Roesler et al., 2001). In addition, similarly to what is observed in experiments using classic CFC (Young et al., 1994), IA training can be experimentally divided into two components: (1) context learning, which depends on *N*-methyl-D-aspartate (NMDA) glutamate receptors in the dorsal hippocampus, and (2) context-footshock association, which is impaired by NMDA receptor blockade in the basolateral amygdala (BLA) but not in the hippocampus (Roesler et al., 1998, 2003).

According to the established view of multiple memory systems, one could assume that the dorsal striatum would be important for the processing of memories for tasks based on procedural learning, but not for the initial learning of single-trial fear-motivated tasks. However, evidence dating back from the 1960s suggests that the striatum may play a more general role in memory formation in tasks including IA. Permanent lesions or reversible inactivation of the dorsal striatum, or pharmacological manipulation of specific striatal neurochemical systems including cholinergic receptors were shown to affect the formation of memory for single-trial IA learning (Kirkby and Kimble, 1968; Haycock et al., 1973; Prado-Alcalá et al., 1975, 1980). More recently, Quirarte and colleagues reported that infusion of the glucocorticoid corticosterone into the dorsal striatum enhanced consolidation of IA in rats (Medina et al., 2007). Glucocorticoids, acting on neuronal glucocorticoid receptors (GRs) in brain areas including the dorsal hippocampus and the BLA, are among the main endogenous modulatory systems involved in enhancing the consolidation of memories associated with emotionally arousing events (for reviews, see Roozendaal, 2000; de Quervain et al., 2009). Somewhat surprisingly, intra-striatal glucocorticoid administration failed to affect memory when contextual and footshock components of IA training were dissociated, suggesting that IA might involve instrumental memory aspects that are selectively regulated by striatal GRs (Medina et al., 2007). An alternative possibility would be that GRs in the striatum modulate specific aspects of IA related to the integration between context and footshock that are absent when the two components are presented separately in distinct training trials.

In a study published in *Frontiers in Behavioral Neuroscience* (volume 6, article 33), Quirarte and colleagues (Sánchez-Resendis et al., 2012) extend those findings by showing a novel functional interaction between GRs and cholinergic receptors within the dorsal striatum that influences IA consolidation. First, they found that an infusion of the muscarinic cholinergic receptor (mAchR) agonist oxotremorine into the dorsal striatum after training enhanced retention of memory for single-trial IA. They then went on to show that the oxotremorine-induced memory enhancement was blocked by either systemic or intra-striatal administration of glucocorticoid signaling inhibitors. Finally, the memory-enhancing effect of intra-striatal corticosterone was blocked by co-infusion of the mAchR antagonist scopolamine. Together, these results indicate that two-way functional interactions between GRs and mAchRs within the dorsal striatum regulate the consolidation of memory for single-training IA.

Some aspects of the findings reported by Sánchez-Resendis et al. (2012) can be highlighted. First, they provide further evidence for an important role of the dorsal striatum in the modulation not only of typical procedural or habit memories, but also of consolidation of a memory for a single-trial, fear-motivated, hippocampus dependent type of conditioning. It would be interesting to examine whether similar effects of striatal manipulation are found when IA retention is measured after a training protocol that certainly does not include instrumental or procedural responses (i.e., when the animals, without having received context preexposure, are put directly into the footshock compartment during training, rather than stepping down or through the footshock area; Roesler et al., 2001). These experiments could help clarify whether the striatum modulates procedural and instrumental aspects of IA training.

Second, this study reveals a novel interaction between GRs and mAchRs within the striatum in modulating memory. A requirement of cholinergic receptors for GR-induced enhancement of memory consolidation had been previously shown in the BLA (Power et al., 2000). It is possible that the dorsal striatum, which has connections with both the BLA and the hippocampus, shares more memory-modulatory functions and mechanisms with these areas than previously thought. From a translational perspective, understanding how neuromodulatory systems within the striatum regulate memory formation might have implications for research on the neuropsychiatric aspects, including cognitive impairments, of neurodegenerative disorders that involve abnormalities in striatal function, such as Huntington’s and Parkinson’s diseases.
